# Implementation of a neuromuscular clinical trial network: a rare disease model for enhancing clinical trial readiness, capacity, and access in Canada

**DOI:** 10.1186/s13023-026-04393-4

**Published:** 2026-05-26

**Authors:** Kerri Lynn Schellenberg, Homira Osman, Maria Masnata, Rhiannon Hicks, Corinne Kagan, Ana Stosic, Stacey Lintern, Erin Beattie, Hanns Lochmuller, Craig Campbell, Jean K. Mah

**Affiliations:** 1 Division of Neurology, Department of Medicine, University of Saskatchewan, Saskatoon, SK Canada; 2 Muscular Dystrophy Canada, Toronto, Canada; 3 Department of Pediatrics, London Health Sciences Centre, Children’s Health Research Institute, Western University, London, Canada; 4 The Neuromuscular Disease Network for Canada, Ottawa, Canada; 5 Precision Child Health, Hospital for Sick Children, Toronto, ON Canada; 6 Division of Neurology and Program for Genetics and Genome Biology, Hospital for Sick Children, Toronto, ON Canada; 7 Children’s Hospital of Eastern Ontario Research Institute, Ottawa, Canada; 8 Division of Neurology, Department of Medicine, The Ottawa Hospital, Ottawa, Canada; 9 Brain and Mind Research Institute, University of Ottawa, Ottawa, Canada; 10 Division of Neurology, Department of Pediatrics, Cumming School of Medicine, Alberta Children’s Hospital Research Institute, University of Calgary, Calgary, AB Canada

**Keywords:** Rare disease, Clinical trials network, Clinical trials, Community of practice, Neuromuscular

## Abstract

**Background:**

Rare diseases affect over 300 million people globally, yet clinical trial conduct in rare disease populations remains complex due to small patient numbers, geographic dispersion, heterogeneous phenotypes, and limited trial infrastructure. Neuromuscular diseases (NMDs) exemplify these challenges. Coordinated trial networks have emerged as a strategy to improve feasibility, access, and trial performance.

**Methods:**

Guided by the Consolidated Framework for Implementation Research (CFIR), we conducted a multi-phase qualitative needs assessment involving neuromuscular investigators and clinical trial personnel across Canada (*n* = 34) and clinical trial personnel (*n* = 16) across Canada. Data were analyzed using conventional qualitative content analysis to identify barriers, facilitators, and capacity-building needs informing the early implementation of a Canadian Neuromuscular Clinical Trial Network (CTN).

**Results:**

Key barriers included burdensome feasibility processes, limited workforce capacity, lack of centralized trial visibility, and fragmented coordination across stakeholders. Trial personnel highlighted unmet needs in disease-specific training, mentorship, peer networking, and workforce sustainability. Early Neuromuscular CTN strategies, including centralized feasibility triage, a national Community of Practice, one-on-one operational monitoring, mentorship, and alignment with patient and registry infrastructures directly addressed these challenges.

**Conclusion:**

The Neuromuscular CTN describes a pragmatic and potentially transferable model for strengthening rare disease clinical trial ecosystems. By ensuring trials not only occur but are optimally supported and performed, coordinated networks can enhance trial readiness, equity of access, and long-term sustainability in rare disease research.

**Clinical trial number:**

Not applicable.

**Supplementary Information:**

The online version contains supplementary material available at 10.1186/s13023-026-04393-4.

## Background

Rare diseases, defined individually by low prevalence but collectively affecting more than 300 million people worldwide, pose persistent challenges for clinical research and therapeutic development [1]. Although over 7,000 rare diseases have been identified, fewer than 5% have an approved disease-modifying treatment [2]. Clinical trials in rare diseases are uniquely complex due to small and geographically dispersed patient populations, phenotypic heterogeneity, limited natural history data, and high per-participant trial costs, all of which complicate study design, feasibility, and recruitment [3].

Furthermore, rare disease clinical trials often serve functions that extend beyond drug evaluation. For many patients and families, participation provides access to specialized expertise, standardized assessments, and close clinical monitoring, and in some cases represents the only opportunity to receive an investigational therapy. Importantly, studies consistently show that people living with rare diseases are motivated to participate in clinical trials not only for potential personal benefit but also by a strong desire to advance scientific knowledge and contribute to treatment development for future generations and the broader patient community [4, 5].

Neuromuscular diseases (NMDs) represent a large and diverse group of rare and ultra-rare conditions, encompassing more than 600 distinct disorders [6]. Common NMDs, including Duchenne muscular dystrophy (DMD), spinal muscular atrophy (SMA), myasthenia gravis (MG), Charcot-Marie-Tooth disease (CMT), Myotonic dystrophy (DM), and inherited muscular dystrophies and neuropathies are typically progressive, multisystem disorders characterized by declining motor function, respiratory insufficiency, cardiomyopathy, and reduced life expectancy. For people living with NMDs, these trajectories result in lifelong disability, substantial caregiving burden, and significant psychosocial and economic impact.

Over the past decade, advances in molecular genetics, antisense oligonucleotides, gene replacement strategies, enzyme replacement therapies, and immune-modulating agents have driven a rapid expansion of clinical trials and regulatory approvals across several NMDs [7]. Despite this progress, access to approved therapies remains uneven internationally, and in countries such as Canada, patients may experience prolonged delays between regulatory approval, health technology assessment, reimbursement decisions, and real-world access. As a result, clinical trials remain a critical pathway for early access to innovation and specialized care, particularly in rare diseases where treatment options are limited.

Historically, Canada has been less frequently selected for clinical trials than the United States and Europe. This reflects several structural and logistical considerations. While geographic dispersion can pose operational challenges, similar distribution issues exist in other large jurisdictions such as the United States. More influential factors likely include market size and regulatory structure. Pharmaceutical sponsors often prioritize jurisdictions with larger patient populations and unified regulatory pathways, such as the United States under the U.S. Food and Drug Administration (FDA) or the European Union under the European Medicines Agency (EMA). In contrast, Canada’s smaller market and additional coordination across provincial health systems, ethics review processes, and institutional contracting requirements can contribute to longer trial start-up timelines. Together, these factors have historically limited Canada’s competitiveness in multinational trial selection and may contribute to delayed access to investigational therapies and, downstream, approved treatments for Canadian patients [8, 9].

In this rare disease context, however, Canada offers a solid environment for clinical trial implementation. Over the past five years, the number of neuromuscular and rare disease clinical trials initiated in Canada has increased substantially, reflecting the global growth in therapeutic development, but also intentional positioning that Canada has made to be more comparable with other established jurisdictions. Rather than a fragmented, competitive trial landscape, neuromuscular care in Canada is characterized by a highly collaborative community of expert clinicians, specialized multidisciplinary centres, and national infrastructures that support coordination and data generation. In addition, Canada’s publicly funded health system supports relatively standardized access to specialized neuromuscular care and diagnostic services, including genetic testing, for much of the population. Compared with some jurisdictions where access to specialized care or diagnostic testing may be limited by financial barriers, this more equitable access can support earlier diagnosis, consistent clinical follow-up, and improved readiness for clinical trial participation. Compared with some jurisdictions where access to specialized care or diagnostic testing may be limited by financial barriers, this more equitable access can support earlier diagnosis, consistent clinical follow-up, and improved readiness for clinical trial participation. Key assets include the Canadian Pediatric Neuromuscular Group (CPNG) (https://www.cpng.ca/), a national collaborative of pediatric neuromuscular specialists; the Canadian Neuromuscular Disease Registry (CNDR) [10], which captures standardized clinical and genetic data across neuromuscular conditions; and engaged disease community organizations such as Muscular Dystrophy Canada (MDC), the national organization supporting people affected by neuromuscular diseases through programs and services, research investment, advocacy, and patient engagement across a broad spectrum of conditions. Building on this established foundation, the Neuromuscular Disease Network for Canada (NMD4C) (https://neuromuscularnetwork.ca/) was established in 2020 through joint funding from the Canadian Institutes of Health Research (CIHR) and MDC. During its initial mandate, NMD4C identified fragmentation in trial coordination, limited visibility of national trial capacity, and unmet workforce needs as barriers to optimal trial conduct. In response, NMD4C launched the Neuromuscular Clinical Trial Network (CTN) in 2024.

In this paper, we describe the rationale, early implementation strategies, and outcomes of the Canadian Neuromuscular CTN. Using neuromuscular diseases as an exemplar, we demonstrate how coordinated national infrastructure can ensure that rare disease clinical trials not only occur, but thrive – performing efficiently, equitably, and in a manner that fully reflects the expertise of participating centres.

## Methods

### Study design and conceptual framework

The development and early optimization of the Neuromuscular CTN were guided by the Consolidated Framework for Implementation Research (CFIR) [11]. CFIR was selected because it is a widely used, theory-informed implementation framework specifically designed to examine the introduction of complex interventions within real-world health systems and to account for the multilevel factors that influence implementation success [12]. Recent implementation-science literature highlights CFIR’s utility for large, system-level initiatives that span multiple organizations, professional groups, and jurisdictions, where contextual variation and interaction between stakeholders are central determinants of outcomes [13].

In rare disease clinical trials, feasibility, workforce capacity, institutional readiness, and external system constraints often intersect. CFIR offers a pragmatic structure to systematically explore determinants across intervention characteristics, outer setting (e.g., regulatory and sponsor environments), inner setting (e.g., institutional capacity and workflows), characteristics of individuals (e.g., investigator and coordinator experience), and implementation processes. Consistent with contemporary guidance on flexible framework application, CFIR was used as a sensitizing framework rather than a prescriptive coding scheme, informing the development of interview guides and the analytic approach. This allowed for inductive identification of barriers, facilitators, and capacity-building opportunities, while ensuring that findings were interpreted within a coherent, multilevel implementation lens. Themes were mapped to CFIR domains during the analytic process.

### Stakeholder identification and recruitment

Participants were identified using purposive sampling. Eligible principal investigators (PIs) were neuromuscular specialists in Canada who had participated in at least one neuromuscular clinical trial in the previous decade or were recognized as having trial readiness. For the purposes of this study, trial readiness was defined as having the clinical and research infrastructure, trained personnel, and prior experience or demonstrated capacity to conduct clinical trials, as well as recognition within the neuromuscular clinical and research community as being capable of supporting trial participation. Clinical trial coordinators and management staff were identified through participating neuromuscular centres and were eligible if they had direct operational involvement in trial implementation.

### Stakeholder outreach and needs assessment

In the initial phase, email invitations were sent to 43 neuromuscular PIs across Canada, representing 12 pediatric and 14 adult centres, identified based on recent clinical trial activity or demonstrated trial readiness. These centres represent the majority of neuromuscular clinical trial-active sites in Canada. While there is no single comprehensive list of all neuromuscular centres nationally, it is estimated that approximately 25–30 centres provide specialized neuromuscular care across Canada. The centres included in this study were identified based on prior clinical trial activity or recognized trial readiness through established national networks, thereby reflecting sites most actively engaged in or positioned to participate in clinical trials.

These invitations aimed to assess interest in participating in the Neuromuscular CTN and to gather preliminary insights on barriers related to industry engagement, trial feasibility, and administrative burden. Thirty-four responses were collected via email, with nine invited PIs not responding to the initial outreach; reasons for non-response were not systematically collected. From the respondents, a subset of PIs (*n* = 7) participated in follow-up one-on-one virtual meetings. Participants for these meetings were purposively selected from respondents to capture a range of clinical trial experience, institutional contexts, and geographic representation across participating centres. These participants were not the only respondents available but were selected to ensure diversity of perspectives relevant to neuromuscular clinical trial implementation.

A more structured assessment followed with semi-structured interviews of clinical trial personnel. Eligible participants were identified through publicly listed neuromuscular clinical trials on ClinicalTrials.gov, which indicated approximately 19 individuals involved in clinical trial coordination or management across neuromuscular Canadian centres, including 7 adult centres, 10 pediatric centres, and 1 combined adult-pediatric centre. All identified personnel were invited to participate, and 16 clinical trial personnel (including clinical research coordinators, research nurses, and trial managers) from 13 centres ultimately participated in the interviews. Interview guides were developed iteratively based on PI feedback and consultations with industry sponsors active in neuromuscular trials. All interviews were audio-recorded with participant consent and transcribed verbatim to ensure accuracy of data capture. Interviews were conducted by two members of the research team (HO, MM), including the Clinical Trial Network Manager and a program lead. Both interviewers have formal training in qualitative research and substantive expertise in health systems implementation and neuromuscular clinical research. Both were actively involved in the development and implementation of the Neuromuscular Clinical Trial Network.

To enhance methodological rigor and address potential reflexivity-related bias arising from their dual roles, interviews were guided by a structured semi-structured interview guide, and analytic procedures incorporated independent coding and consensus-based theme refinement.

### Qualitative analysis

Data from email responses, meeting notes, and interview quotations were analyzed using conventional qualitative content analysis, an inductive analytic approach commonly used to identify patterns and themes within textual data when prior theory or literature on a phenomenon is limited [14]. Two authors (HO, MM) independently reviewed the data to generate initial codes reflecting emerging concepts. Codes were subsequently compared, and discrepancies were resolved through structured discussion until consensus was reached. Coding was conducted manually using an inductive approach, without the use of qualitative data analysis software.

The codes were then organized into broader categories and interpreted using the Consolidated Framework for Implementation Research (CFIR) as an analytic lens to examine multilevel determinants influencing neuromuscular clinical trial conduct in Canada. Through iterative discussion among the research team, themes were refined to reflect key barriers, facilitators, and capacity-building needs relevant to neuromuscular clinical trial implementation. Data collection and analysis continued until thematic sufficiency was reached, with no new major themes identified in later interviews. The study was conducted and reported in accordance with the Consolidated Criteria for Reporting Qualitative Research (COREQ).

### Ethics considerations

This project involved consultation with neuromuscular PIs and clinical trial personnel regarding operational aspects of clinical trial conduct in Canada as part of the development of the Neuromuscular CTN. The activities focused on gathering perspectives on trial processes, workforce capacity, and operational challenges and did not involve patient participants or the collection of personal health information. The work was conducted as part of network development and program implementation. In accordance with institutional guidance, the project was considered program development and did not require formal ethics review.

## Results

### Mission and scope of the neuromuscular CTN

The Neuromuscular CTN was established to strengthen neuromuscular clinical trial readiness and performance across Canada by addressing operational, workforce, and coordination challenges common to rare disease research. The Neuromuscular CTN aims to increase the number of neuromuscular clinical trials conducted in Canada, enhance national trial capacity, and expand equitable opportunities for patient participation through centralized coordination, mentorship, and integration with existing national infrastructures.

Findings from the multi-phase needs assessment directly informed the operational priorities and early implementation strategies of the Neuromuscular CTN. Results are presented across three sections: (1) perspectives of neuromuscular principal PIs, (2) perspectives of clinical trial personnel, and (3) early Neuromuscular CTN implementation strategies aligned to identified needs.

## PI perspectives on neuromuscular clinical trial conduct

PIs described several positive aspects of participating in neuromuscular clinical trials. Many highlighted the professional satisfaction associated with contributing to therapeutic innovation in conditions where treatment options have historically been limited. Clinical trials were also viewed as an important mechanism for providing patients and families with access to specialized expertise, enhanced clinical monitoring, and emerging therapies. Several PIs noted that trial participation strengthens clinician–patient partnerships and allows clinicians to actively contribute to improving care and future treatment options for the neuromuscular community.

Despite these motivations, PIs consistently identified structural and operational barriers that limited their ability to engage in clinical trials. Analysis of PI input revealed four interrelated themes related to barriers and system-level challenges in neuromuscular clinical trial conduct.

### Burdensome feasibility processes and excessive sponsor outreach

PIs consistently described being inundated with direct outreach from pharmaceutical sponsors and contract research organizations requesting feasibility assessments. The feasibility process was widely perceived as time-intensive and duplicative, often involving lengthy questionnaires with limited likelihood of site selection. Many PIs reported that the cumulative administrative burden discouraged engagement, particularly when balanced against clinical responsibilities and ongoing trials.

*This burden was reflected by participants, with one noting, Feasibility assessments are lengthy and often duplicated, and with limited chances of site selection, it becomes difficult to justify the time required alongside clinical responsibilities. (Principal investigator)*

### Resource constraints and workload saturation

PIs emphasized that neuromuscular clinics typically operate with small, highly specialized teams managing complex patient populations. Limited clinical research personnel capacity necessitated careful selectivity in deciding which trials to activate, often leading to missed opportunities. Decisions were frequently driven by feasibility and anticipated workload rather than patient need alone. Participants also highlighted infrastructure limitations that constrained trial participation, with one clinical trial coordinator noting, *“We don’t always have the infrastructure to support research…clinic space is shared with standard care, and we have to fit research visits around everything else.”* (Clinical trial coordinator).

### Limited visibility of trial opportunities across canada

A lack of centralized visibility of neuromuscular clinical trials in Canada was repeatedly identified as a barrier. PIs at smaller centres and early-career PIs reported fewer opportunities to engage in trials despite relevant expertise. Participants highlighted the absence of a shared, up-to-date overview of active and upcoming trials as a limitation to collaboration and equitable trial participation.

### Fragmentation across networks and infrastructures

PIs described inefficiencies arising from siloed activities among clinician networks, patient organizations, and disease registries. Redundant outreach and uncoordinated trial notifications increased workload without improving recruitment, with missed opportunities for collaboration due to limited trial activation and patient referral.

## Clinical trial personnel perspectives on capacity and sustainability

Semi-structured interviews were conducted with 16 clinical trial coordinators and clinical trial management staff from 13 neuromuscular centres across Canada. Participants provided detailed insight into operational challenges, workforce needs, and opportunities for capacity-building. Analysis of trial personnel input revealed four interrelated themes.

### Need for disease-specific training and professional development

Clinical trial personnel expressed a strong desire to deepen their understanding of neuromuscular diseases beyond protocol-specific training. Many reported dedicating personal time to learning about disease mechanisms, progression, and emerging therapies, often motivated by mentorship from engaged PIs. Participants identified a need for accessible, multimodal educational opportunities, including journal clubs, disease-focused meetings, and centralized, unbiased scientific updates independent of sponsor materials. A participant emphasized: *“A lot of what we learn comes from other coordinators and hands-on experience rather than formal training.”* (Clinical trial coordinator).

### Strong interest in peer networking and shared best practices

Participants consistently emphasized the value of connecting with peers across institutions to share operational experiences and problem-solve common challenges. While virtual meetings were viewed as essential for accessibility, there was strong interest in periodic in-person gatherings dedicated specifically to neuromuscular clinical trial personnel, distinct from sponsor-led PI meetings, to foster mentorship and collaboration. The value of cross-site collaboration was consistently emphasized, with one participant noting, *“Knowing that there are people at other sites you can reach out to for support or to share best practices would be incredibly valuable.”* (Clinical trial manager).

### Absence of a centralized source of clinical trial information

Clinical trial personnel highlighted the lack of a reliable, centralized source of information on neuromuscular clinical trials conducted in Canada. Participants expressed a desire to easily identify where trials were active, recruitment status, and site contacts, as well as to receive clear, centralized updates on investigational therapies and Health Canada approvals. A neurologist from a lower-volume clinical trial site emphasized this limitation: *“There isn’t a clear way to know which trials are running at different sites or who is recruiting, which makes coordination and collaboration more difficult.”* (Principal investigator).

### Workforce sustainability and operational barriers

Several systemic barriers to trial efficiency and workforce sustainability were identified. High staff turnover was commonly attributed to short-term contracts, comparatively low salaries, and limited workplace flexibility. Knowledge gaps varied by experience level, with junior coordinators seeking foundational skills (e.g., budget and contract negotiation), while senior staff focused on process optimization. Fragmented research ethics and legal contract review processes were also cited as a major contributor to prolonged trial start-up timelines. The complexity of trial execution and staffing demands was also emphasized, with one participant describing, *“Getting a trial up and running involves many steps across different departments, and staffing those intensive study periods especially for early-phase trials is a real challenge.”* (Clinical trial coordinator).

### Early neuromuscular CTN implementation strategies aligned to identified needs

Findings from the needs assessment directly informed early Neuromuscular CTN implementation strategies (Table 1). To address burdensome feasibility processes and excessive sponsor outreach, the Neuromuscular CTN established a centralized feasibility triage model. When approached by sponsors, the Neuromuscular CTN conducts an initial high-level protocol review summarizing key requirements, anticipated resource demands, and potential operational challenges. This information is shared with relevant PIs based on site capabilities and patient populations, reducing duplicative outreach and supporting more efficient decision-making. Where interest is confirmed, the Neuromuscular CTN facilitates progression to formal feasibility, including execution of confidentiality disclosure agreements between sponsors and participating institutions. In parallel, the Neuromuscular CTN supports early mentorship and peer consultation, enabling less-experienced or lower-volume sites to seek guidance from experienced trialists when assessing feasibility and readiness.

**Table 1:** Alignment of identified barriers, facilitators, and CTN strategies across CFIR domains

CFIR Domain	Barriers and Facilitators Identified	Neuromuscular CTN Strategies
Intervention Characteristics	High complexity of neuromuscular trials; burdensome and duplicative feasibility processes	Centralized feasibility triage and protocol review; staged progression to formal feasibility
Outer Setting	Fragmented sponsor outreach; limited visibility of Canadian trial capacity; geographically dispersed patient populations	Single entry point for sponsors; national clinical trial database; articulation of Canada’s value proposition
Inner Setting	Variable institutional capacity; small, overstretched clinic teams; workflow heterogeneity	Site-specific feasibility matching; operational check-ins; cross-site problem-solving
Characteristics of Individuals	Variable PI and coordinator experience; strong motivation but unmet training needs	Provide mentorship; CoP; tailored capacity-building
Implementation Process	Fragmented coordination across stakeholders; delayed identification of operational challenges	Continuous one-on-one operational monitoring; integration with patient organizations and registries

Abbreviations: CFIR = Consolidated Framework for Implementation Research; CoP = Community of Practice; CTN = Clinical Trial Network

To address resource constraints, professional development needs, peer networking gaps, and workforce sustainability, the Neuromuscular CTN established a national Community of Practice (CoP) based on the Wenger-Trayner Community of Practice framework [15].This framework emphasizes shared learning, peer leadership, and progressive participation among members with varying levels of experience. The Neuromuscular CTN CoP brings together clinical research coordinators, trial managers, research nurses, and PIs from 21 institutions across Canada and convenes through structured bimonthly virtual sessions and semi-annual open forum (https://neuromuscularnetwork.ca/pillar-3-clinical-practice-research/community-of-practice/).

CoP activities focus on practical, experience-based learning related to trial operations, including audit readiness, protocol amendments, trial start-up processes, and cross-jurisdictional regulatory and operational challenges. The CoP is supported by three designated champions (RH, NM, AS), highly experienced clinical trial professionals who serve as peer educators and facilitators, providing continuity, subject-matter expertise, and leadership across sessions. In addition to group-based learning, the Neuromuscular CTN supports structured one-on-one and peer-to-peer mentorship, pairing junior PIs and clinical trial personnel with more experienced colleagues in the same roles who function as peer mentors and champions. This approach was designed to build confidence, foster leadership development, and support long-term trial workforce capacity.

To ensure ongoing alignment to site-level challenges, the Neuromuscular CTN manager (MM) conducts regular one-on-one operational check-ins with participating centres. These consultations monitor workload, staffing changes, sponsor interactions, regulatory delays, and emerging barriers, enabling proactive, tailored support rather than retrospective problem-solving.

To address limited visibility of trial opportunities, the Neuromuscular CTN developed and maintains a national neuromuscular clinical trial database, updated monthly with information provided directly by clinical teams (https://neuromuscularnetwork.ca/pillar-2-clinical-research/clinical-trial-network/clinical-trial-database/#database). The database includes trial status, participating sites, PIs, and coordinator contacts and serves as a shared national resource for collaboration and referral. Quarterly, non-industry updates summarizing neuromuscular clinical trials and recent Health Canada approvals are disseminated to Neuromuscular CTN members.

Finally, to reduce fragmentation across networks and infrastructures, the Neuromuscular CTN maintains close coordination with MDC as the primary national pathway for patient engagement and with the CNDR for alignment on clinical data and recruitment pathways. This coordinated approach minimizes duplicative outreach and supports coherent, patient-centred trial communication across Canada.

## Discussion

This study describes the early implementation of a national clinical trial network designed to address barriers to neuromuscular clinical trial conduct identified directly by neuromuscular PIs and clinical trial personnel across Canada. Through a multi-phase needs assessment, we found that limitations in neuromuscular trial activity were not driven by a lack of clinical expertise or investigator interest, but rather by structural and operational challenges including fragmented communication channels, burdensome feasibility processes, workforce constraints, and limited national visibility of trial opportunities. The early implementation strategies of the Neuromuscular CTN including centralized feasibility triage, facilitation of peer mentorship opportunities, a national CoP, ongoing site-level operational monitoring, and integration with existing patient organizations and registry infrastructures were intentionally designed in response to these findings. Together, these results underscore that in rare diseases where clinical trial capacity is inherently constrained, coordinated national infrastructure can play a critical role in translating scientific and therapeutic opportunities into effective and equitable trial delivery.

These findings align with rare disease literature demonstrating that clinical trials in small populations are uniquely challenged by limited patient numbers, geographic dispersion, phenotypic heterogeneity, incomplete natural history data, and difficulties identifying sensitive and clinically meaningful endpoints [3]. Such constraints complicate trial design, power analysis, feasibility assessment, and recruitment, making rare disease trial ecosystems particularly dependent on coordination, shared expertise, and sustained infrastructure. Without these supports, even scientifically compelling trials risk delayed activation, under-enrolment, or suboptimal execution.

International experience provides strong evidence that coordinated CTNs can mitigate these challenges. In Europe, the TREAT-NMD Alliance (https://www.treat-nmd.org/) established a comprehensive infrastructure combining expert care and trial sites, standardized outcome measures, and disease registries, directly supporting the successful conduct of multiple neuromuscular clinical trials and subsequent regulatory approvals, including therapies for DMD and SMA [16, 17]. Similarly, EURO-NMD has demonstrated that structured collaboration among expert centres improves trial readiness, harmonizes care and research practices, and facilitates cross-border recruitment in neuromuscular diseases [18].

In parallel with these international initiatives, tools have also been developed to improve visibility of clinical trial capacity across neuromuscular centres. The Care and Trial Site Registry (CTSR), developed through the TREAT-NMD and EURO-NMD networks, provides a searchable platform where neuromuscular centres can maintain standardized profiles describing their clinical expertise, infrastructure, patient populations, and trial experience [16]. Such platforms are intended to facilitate trial feasibility assessments and enable industry sponsors and collaborators to efficiently identify appropriate sites for clinical research. Canadian neuromuscular centres have participated in CTSR; however, consistent updating of registry profiles and routine use of the platform have proven challenging. Despite efforts by the Canadian Neuromuscular CTN to encourage centres to maintain profiles and promote the tool to sponsors, uptake has been variable. Unlike some European contexts where CTSR operates alongside integrated patient registries and coordinated trial networks, alignment between CTSR and Canadian data infrastructures such as the CNDR has been more limited. These experiences highlight that while technical platforms can support trial coordination, their effectiveness depends on sustained engagement, integration with existing data systems, and active network governance.

In the United States, the NIH-funded Rare Diseases Clinical Research Network (RDCRN) offers further evidence of the value of coordinated trial infrastructure. The RDCRN has been shown to accelerate recruitment, improve data quality, enhance patient engagement, and contribute to multiple therapeutic advances across rare disease domains through sustained, network-based collaboration [19]. Collectively, these models demonstrate that rare disease trials succeed not solely through scientific innovation, but through deliberate investment in coordination, workforce development, and shared operational capacity.

Collectively, these international models underscore that successful rare disease trial ecosystems depend on coordinated infrastructure that links site identification, data and registry assets, and workforce capacity, alongside active governance that sustains engagement over time.

Within Canada, national and provincial initiatives support complementary aspects of rare disease research and clinical trial delivery. RareKids-CAN (https://www.rarekidscan.com/) is a disease-agnostic pediatric rare disease clinical trial network that brings together patients, families, PIs, research networks, industry collaborators, and regulatory and health system stakeholders. Its mandate includes accelerating trial design, enhancing feasibility and start-up processes, strengthening site capacity, supporting operational readiness, and facilitating access pathways for novel therapies. At the provincial level, CATALIS Québec (https://catalisquebec.com/en/) functions as a clinical trials acceleration platform that supports feasibility assessment, trial start-up, and health-system coordination to improve the efficiency and readiness of Quebec’s clinical research environment.

The Canadian Rare Diseases Network (https://canadianrdn.ca/) is a national collaborative network dedicated to strengthening rare disease research, care, and data ecosystems across Canada. Its mandate includes supporting integrated research platforms, enhancing data sharing and interoperability, advocating for sustained investment in rare disease infrastructure, and facilitating cross-jurisdictional collaboration among academic, clinical, patient, industry, and policy partners.

Disease-specific national research networks have also demonstrated the value of coordinated collaboration. The Canadian ALS Research Network (CALS, https://als.ca/research/cals/ ) provides an example of a well-established national consortium advancing clinical research in amyotrophic lateral sclerosis (ALS). Coordinated through ALS Canada rather than a single academic institution, CALS supports collaboration among ALS clinics and PIs across Canada. This national structure has helped increase the visibility of Canadian ALS trial sites, facilitate advocacy for expanded participation across centres, and strengthen connectivity among ALS clinicians and researchers to address both clinical and research priorities [20]. The network’s centralized coordination and dedicated management have contributed to the growth of ALS clinical research activity across participating sites.

These initiatives demonstrate that Canada has an emerging ecosystem of clinical research infrastructure; however, neuromuscular trials span a broad set of diseases, age groups, and multidisciplinary models of care, and the neuromuscular trial landscape has historically lacked a single, coordinated mechanism to streamline feasibility interactions, build trial workforce capacity, and provide a shared national view of trial activity across centres. The Neuromuscular CTN was established to operationalize these principles within the neuromuscular trial landscape at a time of rapid growth in therapeutic development and trial activity. While neuromuscular trials would occur in Canada in the absence of a formal network, our findings suggest that without coordination, such trials risk underperforming, overburdening study teams, and failing to fully leverage national expertise. Rather than functioning primarily to generate trials, the Neuromuscular CTN is designed to ensure that existing and emerging trial activity is supported, strategically aligned, and positioned to perform at a level consistent with the expertise of participating centres.

An important consideration for national trial infrastructures is scalability as therapeutic pipelines expand. The operational strategies of the Neuromuscular CTN were intentionally designed to function across different levels of network activity. Key components including centralized feasibility triage, the CoP, shared trial visibility through the national database, and mentorship structures support coordination and knowledge exchange across centres without relying on a fixed number of participating sites or trials. As neuromuscular clinical trial activity increases, these structures can continue to support collaboration and information flow while additional governance approaches such as regional coordination, tiered mentorship models, or structured approaches to operational monitoring may be introduced. Designing the Neuromuscular CTN with scalability in mind helps ensure that the network represents a sustainable national infrastructure capable of supporting a growing ecosystem of neuromuscular clinical trials, sites, and trained personnel.

A distinguishing feature of the Neuromuscular CTN is its emphasis on workforce sustainability, peer learning, and mentorship. Although the rare disease literature frequently emphasizes trial design, endpoints, and recruitment, comparatively less attention has been paid to the roles of clinical trial coordinators, managers, and research nurses, despite evidence that workforce instability and limited professional development opportunities can undermine trial efficiency and continuity [14, 21]. Within the neuromuscular community, mentorship often occurs informally through collaborative networks and shared problem-solving among experienced PIs and trial personnel. The Neuromuscular CTN seeks to build on these existing practices by creating opportunities for structured peer exchange through the CoP and by exploring approaches to better support early-career PIs and emerging clinical trial professionals. Strengthening mentorship pathways, workforce development and collaboration with like-minded organizations will be an important focus of the network as it evolves.

The Neuromuscular CTN’s emphasis on regular, one-on-one operational check-ins further differentiates this model from more static network structures. By enabling early identification of site-level challenges such as staffing changes, sponsor interactions, or regulatory delays, the Neuromuscular CTN supports proactive, tailored interventions rather than retrospective problem-solving. This adaptive implementation approach is particularly relevant in rare diseases, where small disruptions can disproportionately affect trial timelines and capacity.

The Neuromuscular CTN model prioritizes integration with existing patient organizations and disease registries rather than creating parallel infrastructures. Patient organizations have been shown to play a critical role in rare disease clinical trials by supporting feasibility assessment, improving trial awareness and recruitment, enhancing retention, and informing protocol acceptability, particularly in small and heterogeneous populations [22, 23]. The EURORDIS Rare Barometer surveys further indicate that while most people living with rare diseases express willingness to participate in clinical trials, lack of accessible information, limited awareness of ongoing studies, and poor coordination between research initiatives and clinical care remain major barriers to participation [5]. Respondents consistently identify patient organizations as trusted sources of trial information and emphasize the importance of alignment with existing care pathways and registries to reduce duplication and participation burden. By collaborating closely with MDC for trial awareness and participant engagement and with established registries for clinical data, the Neuromuscular CTN minimizes duplication, reduces participant fatigue, and strengthens coherent, patient-centred recruitment pathways.

## Limitations and future directions

This study reflects early implementation experiences and qualitative insights from engaged stakeholders. Participation was limited to individuals who responded to outreach and may reflect a subset of more engaged or trial-active sites. While the number of non-respondents is reported, reasons for non-participation were not systematically collected, which may limit interpretation of perspectives from less-engaged centres.

In addition, the study focused primarily on centres with prior clinical trial experience or recognized trial readiness and may not fully capture barriers faced by sites without established research infrastructure. These perspectives are important for understanding broader system-level inequities in trial access and capacity. The study was not designed to formally compare perspectives between adult and pediatric centres, and potential differences between these settings may not have been fully explored.

Quantitative evaluation of longer-term outcomes, including trial activation timelines, sponsor engagement, and patient enrollment will be important to assess the sustained impact of the Neuromuscular CTN. Future work will focus on evaluating performance metrics, strengthening and scaling mentorship models, and exploring policy-relevant implications for improving trial efficiency and access across rare disease contexts.

## Conclusion

Although the Neuromuscular CTN is in an early phase of implementation, our findings highlight several structural and operational barriers that influence neuromuscular clinical trial conduct in Canada. The implementation strategies described, including centralized feasibility coordination, mentorship, and workforce capacity-building, were developed in direct response to these identified needs and aimed to strengthen national trial readiness and collaboration. Future evaluation will be important to assess the impact of this model on trial activation, workforce sustainability, and patient access to clinical research opportunities.

As therapeutic development in rare diseases continues to accelerate, coordinated national infrastructures such as the Neuromuscular CTN will play an important role in ensuring that clinical trials not only occur but are delivered efficiently, equitably, and in a manner that reflects the expertise of participating centres. The experience of the Neuromuscular CTN may also offer a transferable approach for other rare disease communities seeking to strengthen clinical trial ecosystems and expand access to innovation.

## Electronic Supplementary Material

Below is the link to the electronic supplementary material.

Supplementary Material 1

## Data Availability

Not applicable.

## Abbreviations

ALS: Amyotrophic lateral sclerosis
CALS: Canadian ALS Research Network
CFIR: Consolidated Framework for Implementation Research
CMT: Charcot-Marie-Tooth disease
CNDR: Canadian Neuromuscular Disease Registry
CoP: Community of Practice
COREQ: Consolidated Criteria for Reporting Qualitative Research
CPNG: Canadian Pediatric Neuromuscular Group
CTN: Clinical Trial Network
DMD: Duchenne muscular dystrophy
MDC: Muscular Dystrophy Canada
MG: Myasthenia gravis
NMD4C: Neuromuscular Disease Network for Canada
NMDs: Neuromuscular diseases
PI: Principal investigator
RDCRN: Rare Diseases Clinical Research Network
SMA: Spinal muscular atrophy
