# Supplementary material for: Implementation of a neuromuscular clinical trial network: a rare disease model for enhancing clinical trial readiness, capacity, and access in Canada
Source: Orphanet J Rare Dis. 2026 May 26;21:252. doi: 10.1186/s13023-026-04393-4 (PMC13390125; doi:10.1186/s13023-026-04393-4)
Supplement: Supplementary file 1 — Supplementary Material 1 [file 13023_2026_4393_MOESM1_ESM.docx]

**Semi-Structured Interview Guide for *“Implementation of A Neuromuscular Clinical Trial Network: A Rare Disease Model for Enhancing Clinical Trial Readiness, Capacity, and Access in Canada”***

This semi-structured interview guide was developed to explore experiences, perceived barriers, and capacity-building needs related to neuromuscular clinical trial implementation. Questions were open-ended and used flexibly to allow probing and clarification.

**Section 1: Background and Experience**

- Can you describe your role and experience in neuromuscular clinical trials?
- How would you describe your level of knowledge and understanding of neuromuscular diseases and patient experiences?

**Section 2: Barriers and Challenges**

- What are the most significant challenges or barriers you have encountered in conducting neuromuscular clinical trials?
- Can you describe any operational or system-level challenges that impact trial execution?

**Section 3: Training and Preparedness**

- What aspects of your training or experience have best prepared you for your role in clinical trials?
- Looking back, what additional (non-clinical) training would have been helpful to better prepare you?
- What topics related to clinical trials would you like to learn more about?

**Section 4: Collaboration and Knowledge Exchange**

- Are you involved in training or mentoring others in neuromuscular clinical trials? If so, what topics are typically covered?
- Would you find it helpful to connect with other clinical trial personnel working in neuromuscular diseases? Why or why not?
- What would you hope to gain from connecting with others in similar roles?

**Section 5: Community of Practice and Capacity Building**

- What comes to mind when you think about a national community of practice for neuromuscular clinical trial personnel?
- What elements are important for successful learning, knowledge exchange, and team-based training?

**Section 6: Information Access and Engagement**

- How do you currently stay up to date on neuromuscular diseases and clinical trials?
- Are you involved in other communities of practice or training networks?

**Section 7: Preferences and Final Reflections**

- Do you have a preferred platform for participating in virtual meetings or collaborative hubs (e.g., Zoom, Microsoft Teams, other)?
- Is there anything else you would like to share about your experience with neuromuscular clinical trials?
